# Engineered Exosomes-Based Photothermal Therapy with MRI/CT Imaging Guidance Enhances Anticancer Efficacy through Deep Tumor Nucleus Penetration

**DOI:** 10.3390/pharmaceutics13101593

**Published:** 2021-09-30

**Authors:** Min Yang, Xiaohui Wang, Fang Pu, Ying Liu, Jia Guo, Shuzhuo Chang, Guoying Sun, Yinghua Peng

**Affiliations:** 1 Institute of Special Animal and Plant Sciences, Chinese Academy of Agricultural Sciences, Changchun 130112, China; yangmin01@caas.cn (M.Y.); liuying05@caas.cn (Y.L.); guojia@caas.cn (J.G.); changshuzhuo@caas.cn (S.C.); 2 Laboratory of Chemical Biology, Changchun Institute of Applied Chemistry, Chinese Academy of Sciences, Changchun 130022, China; xiaohui.wang@ciac.ac.cn (X.W.); pufang@ciac.ac.cn (F.P.); 3 School of Chemistry and Life Science, Changchun University of Technology, Changchun 130012, China

**Keywords:** engineered exosomes, carbon dots, nucleus targeting, tumor photothermal therapy, multimodal imaging diagnosis

## Abstract

Exosomes, as natural nanovesicles, have become a spotlight in the field of cancer therapy due to their reduced immunogenicity and ability to overcome physiological barriers. However, the tumor targeting ability of exosomes needs to be improved before its actual application. Herein, a multiple targeted engineered exosomes nanoplatform was constructed through rare earth element Gd and Dy-doped and TAT peptide-modified carbon dots (CDs:Gd,Dy-TAT) encapsulated into RGD peptide engineered exosomes (Exo-RGD), which were used to enhance the effect of cancer imaging diagnosis and photothermal therapy. In vitro and in vivo experiments showed that the resulting CDs:Gd,Dy-TAT@Exo-RGD could effectively accumulate at cancer site with an increased concentration owing to the targeting peptides modification and exosomes encapsulation. The tumor therapy effects of mice treated with CDs:Gd,Dy-TAT@Exo-RGD were heightened compared with mice from the CDs:Gd,Dy control group. After intravenous injection of CDs:Gd,Dy-TAT@Exo-RGD into tumor-bearing mice, the temperature of tumors rose to above 50 °C under NIR irradiation and the localized hyperpyrexia induced by CDs could remarkably ablate tumors. The survival rate of the mice was 100% after 60 days. In addition, the CDs:Gd,Dy-TAT@Exo-RGD exhibited higher MRI/CT imaging contrast enhancement of tumor sites than that of CDs:Gd,Dy. Our study identified that engineered exosomes are a powerful tool for encapsulating multiple agents to enhance cancer theranostic efficiency and provide insight into precise personalized nanomedicine.

## 1. Introduction

Nanoparticles (NPs)-based diagnosis and treatment platforms have presented good therapeutic efficiency in cancer. Many artificial NPs have been constructed for cancer diagnosis and therapy [1,2,3]. However, developing a promising theranostic platform still faces great challenges. First, the use of non-self nanomaterials may cause undesirable immune response. NPs can be recognized by the immune system and cleared out of the body through the liver and kidney in vivo [4,5,6]. To address this problem, biomimetic NPs that integrate the features of natural biomaterials and synthetic nanomaterials have been introduced [7,8]. Second, accurate delivery of theranostic platforms to the targeted cancer cells is essential in order to achieve high therapeutic effect and minimize side effects to normal tissues in vivo. Several targeting ligands, such as aptamers [9], antibodies [10], and peptides [11] have been conjugated to the surface of NPs to specifically recognize tumor cells. However, single-ligand guided delivery may be unsatisfactory due to the complex cancer microenvironment and to inherent limitations such as nonspecific uptake by certain kinds of normal cells and the presence of the receptor saturation phenomenon [12,13]. Multiple targeting strategies can increase recognition capability, enabling more accurate delivery to deep tumor cell nuclei. Additionally, imaging diagnoses such as magnetic resonance imaging (MRI) and X-ray tomography (CT) imaging have been considered as powerful technologies for assisting cancer therapy by detecting the location of tumors, monitoring real-time treatment effects and visualizing drug metabolism in the body [14,15]. Multimodal imaging, which integrates the individual benefits of these technologies into one platform, can provide more accurate diagnostic information [16,17]. Therefore, the construction of an ideal cancer theranostic agent that meets the above challenges is highly desirable.

Exosomes, a class of naturally occurring NPs, provide an opportunity for developing new theranostic platforms. They exhibit several advantages when compared to existing synthetic NPs, such as lower immunogenicity, longer blood circulation times and superior capacity to overcome physiological barriers [18]. Due to their endogenous origin and composition, exosomes can escape from the mononuclear phagocyte system, cross the membrane barrier and diffuse into tumor tissue via the enhanced permeability and retention (EPR) effect [19]. The characteristics of inherent biocompatibility and small size allow exosomes to be used as drug delivery vehicles for tumor diagnosis and therapy. Some exosomes-based NPs have been developed to deliver chemotherapeutic drugs, miRNA and siRNA for cancer therapy [20,21]. However, the actual application of exosomes-based tumor drug delivery systems is seriously restricted by their poor targeting ability. Thus, more efforts must be devoted to the construction of multiple targeting exosomes delivery vehicles for the encapsulation of theranostic agents.

As a type of 0-dimensional carbon nanomaterial, carbon dots (CDs) display excellent solubility, environmental friendliness and simple synthesis methods, and have therefore been explored as biomedical nanomaterials for cancer theranostics [22,23]. According to a previous report, dopamine (the main composition of melanin in the body) as a carbon source can be carbonized to CDs and self-polymerize on the surface of the CDs to form polydopamine (PDA) layers [24], which possess high photothermal efficiency and have been identified as ideal photothermal materials for photothermal cancer therapy (PTT) [25,26,27]. PTT takes advantage of the sensibility of tumor cells to heat in order to induce apoptosis, and can kill tumor cells directly or increase their susceptibility to chemotherapeutics, thus reducing the drug dose or overcoming multi-drug resistance [28,29,30,31]. Therefore, we speculated that exosomes could serve as endogenous vectors to deliver PTT agent CDs to deep tumor tissue, which could enhance the cancer PTT effect, avoid undesirable immune response and acquire excellent biocompatibility (another key factor for a theranostic agent).

Herein, we designed and constructed a multiple targeted delivery nanoplatform based on exosomes and CDs for imaging-guided cancer therapy. The tumor cell targeting peptide RGD was modified on the surface of an exosomes (Exo-RGD) which possessed high affinity to the integrin α_v_β_3_ over-expressed on tumor cells [32], increasing receptor-mediated endocytosis. Then, rare earth element Gd and Dy-doped CDs conjugated with the nucleus-targeting peptide TAT (CDs:Gd,Dy-TAT) were encapsulated into Exo-RGD. The resulting CDs:Gd,Dy-TAT@Exo-RGD could effectively accumulate at tumor sites with an increased concentration, due to the dual ligand peptide modification and exosomes encapsulation. The CDs:Gd,Dy-TAT@Exo-RGD could penetrate to cell nuclei, leading to the burning of tumor cell nuclei and the ablation of tumors in vivo under NIR laser irradiation. The intrinsic characteristics of Gd and Dy endowed the engineered exosomes with MRI and CT imaging properties. Both the cancer imaging diagnosis and the therapeutic efficiency were enhanced due to exosomes encapsulation and multiple targeting.

## 2. Materials and Methods

### 2.1. Materials

GdCl_3_·6H_2_O (99.99%), DyCl_3_·6H_2_O (99.99%), dopamine hydrochloride (DA), dimethyl sulfoxide (DMSO), 3-(4,5-dimethyl-2-thiazolyl)-2,5-diphenyl-2-H-tetrazolium bromide (MTT), 1-ethyl-3-(3-dimethyl-aminopropyl), N-Hydroxy-sulfosuccin-imide (NHS) and carbodiimide hydrochloride (EDC) were obtained from Sigma Aldrich (St. Louis, MO, USA). Calcein-AM and Propidium Iodide (PI) were purchased from Nanjing KeyGen BioTech. (Nanjing, China). RGD functionalized 1,2-dioleoyl-sn-glycero-3-phosphoethanolaminepoly (ethylene glycol)-2000 (DSPE-PEG-RGD) peptide (Arg-Gly-Asp) and TAT peptide (YGRKKRRQRRR) were bought from Nanjing Peptide Biotech. Ltd. (Nanjing, China). Radio immunoprecipitation assay (RIPA) buffer, bicinchoninic acid protein assay reagent (BCA), 4′,6-Diamidino-2-phenylindole (DAPI) and DiI fluorescent probe were obtained from Beyotime Biotech. (Shanghai, China). The CD63, CD81 and TSG101 antibodies were acquired from Abcam (Cambridge, UK).

### 2.2. Cells Culture

4T1 cell line was bought from Shanghai Cell Bank of CAS. HeLa cell line and Raw 264.7 macrophage cell line were nice gifts from Dr. XH Wang (Changchun Applied Chemistry Institution of CAS). HeLa cells were cultured in Dulbecco’s modified Eagle’s medium (DMEM) containing 10% fetal bovine serum (FBS) and 1% penicillin and streptomycin at 37 °C under 5% CO_2_. Raw 264.7 cells and 4T1 cells were incubated in Roswell Park Memorial Institute 1640 (RPMI 1640) under the same conditions. All consumables and media for cell culture were bought from Gibco (Grand Island, NY, USA).

### 2.3. Preparation of CDs:Gd,Dy-TAT

Rare earth element doped CDs were synthesized by hydrothermal method using dopamine as a carbon source. In a typical synthesis, dopamine hydrochloride (0.5 g), GdCl_3_·6H_2_O (38 mg) and DyCl_3_·6H_2_O (40 mg) were added to 15 mL deionized water. The solution was transferred to a 30 mL teflon reaction kettle after stirring for 30 min, and then heated to 180 °C for 8 h. When the kettle was cooled to room temperature, centrifugation (11,000 rpm, 15 min) was conducted to remove the precipitate, then the solution was dialyzed against deionized water (MWCO = 2000). After 24 h dialysis, the obtained solution was filtrated with 0.22 μm filter (Millipore, Billerica, MA, USA). Finally, the resulting CDs:Gd,Dy NPs were lyophilized through a freeze-dryer.

The TAT peptide was connected to the CDs:Gd,Dy by EDC and NHS reaction [33]. First, CDs:Gd,Dy solution (1 mg·mL^−1^) was mixed with 10 mg EDC and 10 mg NHS, and the carboxyl groups were activated for 30 min under room temperature. Then, 12 mg TAT peptide was added followed by stirring for another 12 h. Finally, the solution was dialyzed with deionized water for 24 h for the removal of unreacted TAT peptide, producing CDs:Gd,Dy-TAT NPs.

### 2.4. Preparation of Exo-RGD and CDs:Gd,Dy-TAT@Exo-RGD

Raw 264.7 cells were cultured in RPMI 1640 medium containing 10% exosomes-depleted FBS and 1% penicillin and streptomycin for 24 h. To modify the exosomes with RGD peptide, the culture media was replaced by RPMI 1640 medium containing DSPE-PEG-RGD (100 μg·mL^−1^), then incubated under the same condition for another 24 h. The exosomes were then isolated from the harvested supernatant according to a method as described earlier [34]. In a typical experiment, for the removal of cells and debris, the supernatant was centrifuged at 300× *g* for 10 min, 2000× *g* for 30 min and 10,000× *g* for 30 min at 4 °C, then filtered with 0.22 μm filter. After that, the filtered supernatant was ultracentrifuged at 120,000× *g* for 90 min at 4 °C. The exosomes pellets resuspended in PBS were ultracentrifuged again at 120,000× *g* for 90 min, 4 °C. Finally, the obtained Exo-RGD were resuspended in PBS and stored at −80 °C for further use.

Sonication was introduced to transfer the CDs:Gd,Dy-TAT into the Exo-RGD. CDs:Gd,Dy-TAT (1 mg·mL^−1^, 100 μL) and Exo-RGD (2 mg·mL^−1^ measured by protein content, 100 μL) were reacted according to the following parameters: amplitude 20%, 6 times of 30 s “on/off” cycles within 3 min, and there was a 2 min cooling process in every cycle. To recovery the exosomes membrane, the mixture was incubated at 37 °C for an hour after sonication. Then, centrifugation was used to collect the CDs:Gd,Dy-TAT@Exo-RGD and the obtained solution was stored at −80 °C.

### 2.5. Characterization of CDs:Gd,Dy-TAT@Exo-RGD

Transmission electron microscopy (TEM) were imaged on a H-7650 instrument with 200.00 kV accelerating voltage. 20 μL of the CDs:Gd,Dy, CDs:Gd,Dy-TAT, Exo-RGD or CDs:Gd,Dy-TAT@Exo-RGD was added to a carbon film copper, then the solution was dyed with 2% uranyl acetate. The constituent elements were tested by X-ray photoelectron spectrometry spectra (XPS). Dynamic light scattering (DLS) was conducted for the measuring of zeta potential and hydrodynamic diameter. The NIR absorption performance was carried out with an Enzyme standard instrument. The inductively coupled emission spectrometer (ICP-710ES, Agilentr, Santa Clara, CA, USA) was used to obtain the Gd^3+^ and Dy^3+^ concentrations. The longitudinal relaxation times and transverse relaxation times (*T*_1_ and *T*_2_) of CDs:Gd,Dy, CDs:Gd,Dy-TAT and CDs:Gd,Dy-TAT@Exo-RGD solution were acquired on a 7 T NMR instrument (Bruker III 400 MHz, Karlsruhe, Germany) at 25 °C.

### 2.6. Western Blot

Exo, Exo-RGD or CDs:Gd,Dy-TAT@Exo-RGD in PBS, respectively, was mixed with RIPA buffer, and BCA reagent was applied to obtain the protein concentration. The samples were boiled and separated using 10% sodium dodecylsulfate-polyacrylamide gel electrophoresis (SDS-PAGE) and transferred onto polyvinylidene fluoride (PVDF) membrane. The membrane was blocked with 5% blotting grade blocker nonfat dry milk at room temperature for 60 min to prevent nonspecific binding. The blocked membrane was subsequently incubated with primary antibodies against CD63, CD81and TSG101 at 4 °C overnight. Tris-buffer saline (pH = 7.4) tween (0.05%) (TBST) was introduced to wash the membrane three times (ten min each time). Then, the secondary antibody was co-incubated with the membrane at room temperature for 60 min. The antibody compound adhered on the membrane was visualized and photographed using a chemiluminescence kit after adequate washing.

### 2.7. Photothermal Performance of CDs:Gd,Dy-TAT@Exo-RGD

To evaluate the photothermal conversion efficiency, CDs:Gd,Dy, CDs:Gd,Dy-TAT, Exo-RGD or CDs:Gd,Dy-TAT@Exo-RGD aqueous solution (1 mL) was dropped into a quartz tube, then irradiated with an external NIR laser (Beijing Stone Laser Co. Ltd., Beijing, China). The temperature was recorded using a digital thermometer every 30 s, and the temperature change during the process was monitored using a thermal infrared camera. To investigate the influence of the sample concentration on photothermal effect, different concentrations of the CDs:Gd,Dy-TAT@Exo-RGD aqueous solution (equivalent CDs:Gd,Dy: 0, 50, 100, 200, 400 μg·mL^−1^) were irradiated with the laser at the power density of 1.6 W·cm^−2^. Meanwhile, the CDs:Gd,Dy-TAT@Exo-RGD aqueous solution (equivalent CDs:Gd,Dy: 200 μg·mL^−1^) was irradiated at different laser energy density (0.2, 0.4, 0.8 and 1.6 W·cm^−2^) to study the effect of laser energy density on photothermal performance.

Moreover, the photostability of CDs:Gd,Dy-TAT@Exo-RGD was also measured. Briefly, the temperatures were recorded with five times of “off and on” cycle, and the UV-Vis-NIR absorbance values of 808 nm were also measured after laser irradiation during an hour.

### 2.8. Cytotoxicity and PTT Effect of CDs:Gd,Dy-TAT@Exo-RGD In Vitro

For quantitative cytotoxicity evaluation of CDs:Gd,Dy-TAT@Exo-RGD in vitro, the MTT method was employed to measure the cell viability of 4T1 and HeLa cells. 4T1 and HeLa cells in 96-well plates were co-incubated with CDs:Gd,Dy, CDs:Gd,Dy-TAT, CDs:Gd,Dy@Exo-RGD or CDs:Gd,Dy-TAT@Exo-RGD in different concentrations (equivalent CDs:Gd,Dy: 0, 100, 200, 400 and 800 μg·mL^−1^) for 24 h, then the culture medium was discarded followed by washing three times with PBS. Next, fresh media was refilled and the cells were irradiated under an NIR laser (1.6 W·cm^−2^, 8 min). After laser irradiation, the cells were cultured for another 24 h. The cells treated with PBS and without laser irradiation were set as control. The culture medium containing 5 mg·mL^−1^ of MTT was added to the plates to form formazan after 4 h incubation. The medium was then abandoned, and the forming formazan crystal was dissolved with 150 μL of DMSO at room temperature. Lastly, an enzyme standard instrument was utilized to detect the optical absorbance at 490 nm.

AM and PI dyes were used to stain the living cells and dead cells, respectively. HeLa cells were cultured in 6-well plates and incubated with Exo-RGD, CDs:Gd,Dy, CDs:Gd,Dy-TAT, CDs:Gd,Dy@Exo-RGD or CDs:Gd,Dy-TAT@Exo-RGD for 24 h followed by 808 nm laser irradiation (1.6 W·cm^−2^, 8 min); the cells without irradiation were as control. A mixture solution of AM (2 μM) and PI (8 μM) dye was added to stain the cells. The fluorescence images of the cells were taken via a fluorescence microscope after sufficient washing with PBS.

### 2.9. Cellular Uptake Assay In Vitro

The internalization of CDs:Gd,Dy-TAT@Exo-RGD was evaluated using fluorescence microscopy. HeLa cells were cultured in 6-well plates overnight until the confluency was 80%. After incubation with DiI dyed CDs:Gd,Dy, CDs:Gd,Dy-TAT, CDs:Gd,Dy@Exo-RGD or CDs:Gd,Dy-TAT@Exo-RGD for 24 h and sufficient washing, 300 μL of DAPI (10 μg·mL^−1^) was added to dye the nuclei. The cellular uptake of these NPs was evaluated by fluorescence microscopy.

### 2.10. Animals and Tumor Xenografts

All animals were handled in strict accordance with good animal practices according to the Animal Ethics Procedures and Guidelines of the People’s Republic of China, and the study was approved by The Animal Administration and Ethics Committee of the Institute of Special Animal and Plant Sciences, Chinese Academy of Agricultural Sciences (Permit No. ISAPSAEC-2020-001DC). Eight week old BALB/c mice (female, weighted 20 g, n = 6 for all the in vivo experiments) were bought from Shenyang Changsheng Biology Co. Ltd. (Shenyang, China) The mice were raised in standard cages in sterile conditions with 12 h dark and 12 h light cycle, five per group. The food and water were free access. For 4T1 tumor xenografts, 5 × 10^5^ 4T1 cells were suspended in PBS (100 μL, pH = 7.5) and then implanted into the mice subcutaneously.

### 2.11. In Vivo Tumor Targeting Ability Evaluation

To monitor the tumor targeting ability and biodistribution in vivo, CDs:Gd,Dy, CDs:Gd,Dy-TAT and CDs:Gd,Dy-TAT@Exo-RGD were labeled with NHS-Cy5.5. The tumor-bearing mice were divided into three groups randomly; the labeled NPs were then injected into the mice through the tail vein. Then, in vivo fluorescence images after different time points were obtained with a Bruker In-Vivo FXPRO imaging system (Bruker Magnetic Resonance, Karlsruhe, Germany). The mice were then executed, and the major organs and tumors were collected. Finally, the ex vivo fluorescence images were taken using the same imaging system.

Biodistribution was also assessed by measuring the Gd concentration in the organs of tumor-bearing mice. The mice were administrated with CDs:Gd,Dy, CDs:Gd,Dy-TAT or CDs:Gd,Dy-TAT@Exo-RGD solution through the tail vein (equivalent CDs:Gd,Dy: 15 mg·Gd·kg^−1^·wt, 100 μL). The mice were then executed after different times (6 h, 12 h, 24 h and 7 day) and the major organs, including heart, liver, spleen, lung and kidney as well as the tumors were collected. The samples were added to concentrated nitric acid and dissolved at 200 °C using a digestion instrument. The amount of Gd was measured using ICP-710ES. The biodistribution was assessed by comparing the Gd content per gram of different samples.

### 2.12. Relaxivity and MRI In Vitro and In Vivo

A series of CDs:Gd,Dy, CDs:Gd,Dy-TAT and CDs:Gd,Dy-TAT@Exo-RGD solutions of different Gd and Dy concentrations (Gd/Dy: 0, 0.01, 0.02, 0.04, 0.08, and 0.16 mM) were prepared to measure the longitudinal relaxation values (*r*_1_) and transversal relaxation values (*r*_2_), which were used to evaluate the imaging performance. Relaxation times *T*_1_ and *T*_2_ were measured using a 7 T NMR spectrometer. *r*_1_/*r*_2_ values were the slopes of the lines that were plotted from reciprocal values of relaxation times and Gd^3+^ and Dy^3+^ concentration. As for virtual *T*_1_-MRI and *T*_2_-MRI, different concentration of CDs:Gd,Dy-TAT@Exo-RGD solutions were added into 1.5 mL tubes, then the tubes were imaged through a clinical MRI system (GE Discovery MRI 750 3.0 T, Little Chalfont, Buckinghamshire, UK).

For *T*_1_-MRI and *T*_2_-MRI in vivo, the mice were injected with CDs:Gd,Dy, CDs:Gd,Dy-TAT or CDs:Gd,Dy-TAT@Exo-RGD solution (100 μL) through the tail vein and imaged on a clinical MRI system (GE Discovery MRI 750 3.0 T). For *T*_1_-MRI, the fast recovery spin-echo sequence (FR-FSE) parameters were as following: FOV read = 210 × 210 mm, TE = 21.0 ms, TR = 570 ms, thickness of slice: 1.9 mm. For *T*_2_-MRI, FR-FSE parameters were as following: FOV read = 230 × 230 mm, matrices = 200 × 200, TE = 110 ms, TR = 4000 ms, thickness of slice: 1.9 mm.

### 2.13. In Vitro and In Vivo CT Imaging

A series of different Gd and Dy concentration (Gd and Dy: 1.25, 2.5, 5, 7.5 and 10 mg·mL^−1^) CDs:Gd,Dy-TAT@Exo-RGD solutions were put into 1.5 mL tubes and phantom CT imaging was carried out through Philips Brilliance iCT type 256 instrument. The CT values of hounsfield units (HU) were acquired from the brilliance workspace. Firstly, 100 μL of 10% chloral hydrate solution were injected to anesthetize the mice though the abdominal cavity before CT imaging in vivo. Then 100 μL of CDs:Gd,Dy, CDs:Gd,Dy-TAT or CDs:Gd,Dy-TAT@Exo-RGD (Gd+Dy: 30 mg·kg^−1^·wt) was injected into the mice through the tail vein. Finally, the CT imaging was processed on a Philips Brilliance iCT type 256 instrument with the imaging parameters of 120 KVp, SW: 1.0 mm, FOV: 72 mm and Z: 2.00.

### 2.14. Photothermal Effect of CDs:Gd,Dy-TAT@Exo-RGD In Vivo

The tumor-bearing mice were randomized into five groups for different administrations: (1) intravenously injected with PBS (100 μL); (2) 808 nm laser irradiation only, 10 min; (3) intravenously injected with CDs:Gd,Dy (100 μL, 15 mg·kg^−1^·wt) with a 808 nm laser irradiation, 10 min; (4) intravenously injected with CDs:Gd,Dy-TAT (100 μL, equivalent CDs:Gd,Dy: 15 mg·kg^−1^·wt) with a 808 nm laser irradiation, 10 min; (5) intravenously injected with CDs:Gd,Dy-TAT@Exo-RGD (100 μL, equivalent CDs:Gd,Dy: 15 mg·kg^−1^·wt) with a 808 nm laser irradiation, 10 min. The body weights and tumor volumes were recorded every two days during 21 days, and the tumor volumes were measured per the following formula: length × width^2^/2. A thermal infrared camera (FLIR One, Wilsonville, ORE, USA) was employed for the monitoring of the temperature change of the mice from different groups.

### 2.15. In Vivo Biosafety of CDs:Gd,Dy-TAT@Exo-RGD

Healthy mice were injected intravenously with PBS, CDs:Gd,Dy, CDs:Gd,Dy-TAT, Exo-RGD or CDs:Gd,Dy-TAT@Exo-RGD solution (100 μL, equivalent CDs:Gd,Dy: 15 mg·kg^−1^·wt). After 14 days, the mice were executed and the major organs (heart, liver, spleen, lung and kidney), muscle and brain were harvested for histological assessment. The samples were embedded in paraffin and sliced consecutively into 3 μm thickness after 48 h fixation in 4% paraformaldehyde. Then, the samples were dyed with hematoxylin and eosin (H&E), and the histopathological changes were observed by a microscope. At the same time, behaviors such as drinking, eating, activities, fur color and nervous system were monitored. Moreover, routine blood analysis and blood chemistry tests were performed.

### 2.16. Statistical Analysis

Quantitative results were presented as the mean value ± SD. Statistical analysis was performed using two-way ANOVA and 2-tailed unpaired Student’s *t*-test (SPSS software 12.0, IBM, Armonk, NY, USA; GraphPad Prism 7.0, GraphPad Software, San Diego, CA, USA). A value of *p* < 0.05 was considered significant.

## 3. Results and Discussion

### 3.1. Synthesis and Characterization of CDs:Gd,Dy-TAT@Exo-RGD

**Scheme 1:**
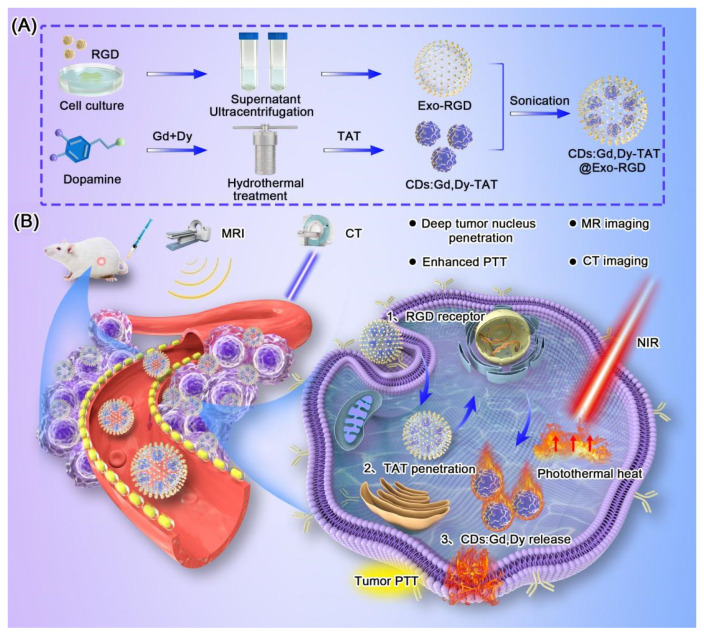
(**A**) Synthesis schematic illustration of CDs:Gd,Dy-TAT@Exo-RGD. (**B**) Schematic illustration of CDs:Gd,Dy-TAT@Exo-RGD as a multiple targeted delivery nanoplatform to enhance tumor photothermal therapy and MR/CT imaging.

**Figure 1:**
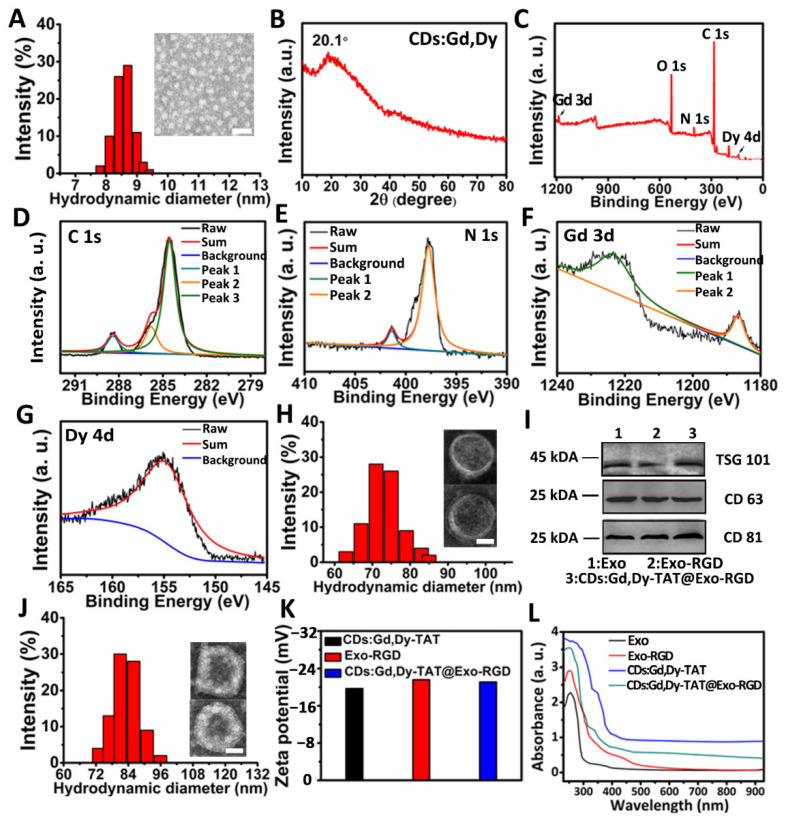
Preparation and characterization of CDs:Gd,Dy-TAT@Exo-RGD. (**A**) Hydrate particle size and representative TEM images (inset) of CDs:Gd,Dy, scale bar: 30 nm. (**B**) XRD pattern of CDs:Gd,Dy. (**C**) XPS spectrum of CDs:Gd, Dy. High resolution XPS spectra of C 1s (**D**), N 1s (**E**), Gd 3d (**F**) and Dy 4d (**G**). (**H**) Hydrate particle size and representative TEM images (inset) of Exo-RGD, scale bar: 30 nm. (**I**) Western blot qualitative analysis of TSG101, CD63 and CD81. (**J**) Hydrate particle size and representative TEM images (inset) of CDs:Gd,Dy-TAT@Exo-RGD, scale bar: 30 nm. (**K**) Zeta potentials analysis of CDs:Gd,Dy-TAT, Exo-RGD and CDs:Gd,Dy-TAT@Exo-RGD. (**L**) UV-Vis-NIR absorption spectra of Exo, Exo-RGD, CDs:Gd, Dy-TAT and CDs:Gd, Dy@Exo-RGD.

Scheme 1 showed the synthesis method and process of the CDs:Gd,Dy-TAT@Exo-RGD. Gd and Dy-doped CDs (CDs:Gd,Dy) were firstly prepared through the hydrothermal approach choosing dopamine as the carbon source. In TEM images, the CDs:Gd, Dy were homogeneous dots with an average size of 7.9 ± 0.5 nm (Figure 1A). The hydrodynamic diameter obtained from dynamic light scattering (DLS) was measured to be about 8.5 nm. There was a slight difference between the size of TEM and DLS, which was due to the difference in the tested conditions (DLS: aqueous sample, TEM: dry sample). The X-ray diffraction (XRD) spectrum was acquired to identify the crystal nature of CDs:Gd,Dy (Figure 1B). There was a broad diffraction peak at around 20.1° due to the highly disordered carbon atoms, similar to the lattice spacing of graphite. X-ray photoelectron spectroscopy (XPS) analysis was conducted to study the elementary composition and the chemical structure of the CDs:Gd,Dy. As shown in the XPS spectrum of full scan (Figure 1C), there were five dominant peaks assigned to C 1s (285.1 eV), O 1s (532.5 eV), N 1s (398.9 eV), Gd 3d (1187.3 eV) and Dy 4d (156.2 eV). The element valence in CDs:Gd,Dy was then analyzed. The C 1s high-resolution XPS spectrum in Figure 1D showed three peaks at 288.4 eV, 285.7 eV and 284.7 eV, which corresponded to the C=O, C–N/C–O and C=C, respectively. In the N 1s high-resolution spectrum (Figure 1E), there were two peaks assigned at 401.5 eV and 398.2 eV, belonging to the N–H and N–C [35]. These facts demonstrated that the obtained CDs:Gd,Dy were functionalized with hydroxyl, carboxylic acid and amino groups, resulting in remarkable solubility in water. Figure 1F showed two characteristic peaks attributed to Gd 3d_3/2_ (1224.5 eV) and Gd 3d_5/2_ (1186.2 eV). The high-resolution spectrum in Figure 1G showed the existence of a peak at 156.1 eV assigned to Dy 4d. Therefore, we concluded that Gd and Dy were successfully incorporated into the CDs.

After modification with a classic cell penetration peptide TAT, which possess strong nucleus targeting properties, the average size of the CDs:Gd,Dy-TAT increased to ~9.0 nm (Figure S1). For the sake of minimizing the effect on the immune system, avoiding potential long-range toxicity in body, and taking ease of production into consideration, the macrophage cell line Raw 264.7 was chosen as a donor to produce exosomes as a drug delivery vehicle [36]. The cells were incubated with culture containing DSPE-PEG-RGD, and the culture supernatant was treated with filtration and differential ultracentrifugation to generate Exo-RGD. In the TEM images (Figure 1H), the vesicles were round-like with clear membrane structure and average size of 70 ± 9 nm. Western blot analysis (Figure 1I) revealed that both Exo and Exo-RGD expressed characteristic membrane proteins of exosomes including TSG101, CD63 and CD81. Finally, the CDs:Gd,Dy-TAT were encapsulated into Exo-RGD by sonication. TEM images (Figure 1J) showed that the structure integrity of exosomes was retained, demonstrating that the sonication treatment did not destroy the exosomes membrane, which ensured the biological function of the exosomes in vivo. The size of the CDs:Gd, Dy-TAT@Exo-RGD was ~79 nm; the slight increase of the diameter may due to the cargoes in the exosomes. TSG101, CD63 and CD81 (the typical marker proteins of exosomes) were all detected in Exo, Exo-RGD and CDs:Gd,Dy-TAT@Exo-RGD (Figure 1I). All the NPs exhibiting these marker proteins revealed that the RGD engineering and the further sonication transition caused negligible influence on the structure and the function of the exosomes. The zeta potential of CDs:Gd,Dy-TAT was −20.0 mV because of the negatively charged TAT peptide (Figure 1K). After being transferred into Exo-RGD, the zeta potential decreased to −21.8 mV, which was similar to that of Exo-RGD (−22.3 mV). The zeta potentials confirmed the successful engineering of the exosomes. The quantity of RGD peptide and TAT peptide on the CDs:Gd, Dy-TAT@Exo-RGD were further measured. On the basis of the absorbance value of FITC on CDs:Gd,Dy-TAT@Exo-RGD, the concentrations of the conjugated RGD and TAT were calculated to be 76 nM and 94 nM, respectively, resulting from the RGD/TAT-FITC absorbance concentration standard curve (Figures S2 and S3). Moreover, the physiological stability of CDs:Gd,Dy-TAT@Exo-RGD was also assessed (Figures S4−S7).

### 3.2. Photothermal Performance and Thermal Imaging of CDs:Gd,Dy-TAT@Exo-RGD

**Figure 2:**
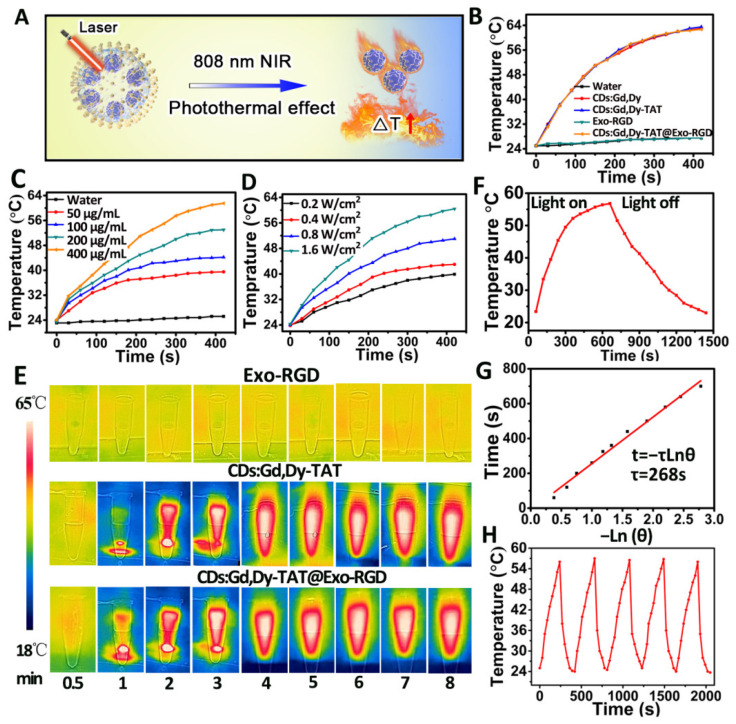
Photothermal conversion performance of CDs:Gd,Dy-TAT@Exo-RGD. (**A**) The schematic illustration of the photothermal effect triggered by NIR laser. (**B**) Temperature change curves of water, CDs:Gd,Dy, CDs:Gd,Dy-TAT, Exo-RGD and CDs:Gd,Dy-TAT@Exo-RGD under NIR laser irradiation (808 nm, 1.6 W·cm^−2^). (**C**) Temperature change curves of CDs:Gd,Dy-TAT@Exo-RGD (50 μg·mL^−1^, 100 μg·mL^−1^, 200 μg·mL^−1^, 400 μg·mL^−1^) under NIR laser irradiation (808 nm, 1.6 W·cm^−2^). (**D**) Temperature change curves of CDs:Gd,Dy-TAT@Exo-RGD at different laser power densities (0.2, 0.4, 0.8, and 1.6 W·cm^−2^). (**E**) Thermal infrared images of Exo-RGD, CDs:Gd,Dy-TAT and CDs:Gd,Dy-TAT@Exo-RGD aqueous solutions under NIR laser irradiation (808 nm, 1.6 W·cm^−2^) at different time points. (**F**) Temperature change curve of CDs:Gd,Dy-TAT@Exo-RGD aqueous solution under NIR laser irradiation (808 nm, 1.6 W·cm^−2^) with laser on and then off after 10 min. (**G**) Time constant of heat transferring measured by the time versus the negative natural logarithm of the temperature. (**H**) Temperature change curves of CDs:Gd,Dy-TAT@Exo-RGD solution under repeated (five times) on and off laser irradiation cycling.

The UV-Vis-NIR absorbance spectra of Exo, Exo-RGD, CDs:Gd,Dy-TAT and CDs:Gd,Dy@Exo-RGD aqueous solution was measured under the same conditions to evaluate photothermal performance. Wide absorbance at 550–850 nm of CDs:Gd,Dy-TAT and CDs:Gd,Dy-TAT@Exo-RGD was found, shown in Figure 1L. We further investigated the photothermal property when the NPs were exposed to NIR laser (1.6 W·cm^−2^, 8 min, Figure 2A); the temperature changes (ΔT) were recorded during the process. The ΔT for CDs:Gd,Dy-TAT@Exo-RGD solution were as follows: ~15 °C, ~20 °C, ~27 °C and ~39 °C for the concentration of 50 μg·mL^−1^, 100 μg·mL^−1^, 200 μg·mL^−1^ and 400 μg·mL^−1^. Similar results were obtained for CDs:Gd,Dy and CDs:Gd,Dy-TAT (Figure 2B,C). The ΔT for water and Exo-RGD remained the minimum change, demonstrating that CDs:Gd,Dy was responsible for the photothermal conversion and that the exosomes loading couldn’t affect the NIR light absorption capacity of the CDs. The CDs:Gd,Dy-TAT@Exo-RGD aqueous solution was then irradiated at different power densities. After irradiation for 8 min, there was a 15 °C temperature elevation for the power density of 0.2 W·cm^−2^, while a higher value of 36 °C was obtained at 1.6 W·cm^−2^, suggesting that temperature elevation of the solution became faster with the increase of laser energy density (Figure 2D). The thermal imaging was also conducted using a thermal infrared camera under NIR laser irradiation. As clearly shown in Figure 2E, with the extension of time, both of the thermal images of the CDs:Gd,Dy-TAT and CDs:Gd,Dy-TAT@Exo-RGD solutions became brighter and brighter. However, the brightness of the Exo-RGD solution showed negligible increase after irradiation for 8 min, which was in accordance with the above results. The photothermal conversion efficiency (η) can assess the capacity of a photo-thermal agent to convert laser energy into heat. Here, the temperature heating curve of the CDs:Gd,Dy-TAT@Exo-RGD aqueous solution was conducted as a series of time under NIR laser irradiation (1.6 W·cm^−2^). The laser power was turned off 10 min later, then the cooling temperature was carefully recorded every 30 s in order to determine the rate of heat transfer from the aqueous solution into its surroundings (Figure 2F,G). Based on this, the η of the CDs:Gd,Dy-TAT@Exo-RGD was calculated to be 44.2%, which was because of the high surface area of the PDA coating on the CDs. Moreover, the TEM image (Figure S8) showed that the exosomes membrane was destroyed by the heat under NIR laser irradiation and the CDs:Gd,Dy-TAT were released to the exterior environment, guaranteeing photothermal efficiency and ensuring continuous photothermal conversion in the tumor. In addition, the photostability of CDs:Gd,Dy-TAT@Exo-RGD was evaluated. The solution was irradiated by NIR laser, then the laser was cycled off and on five times. There was no distinct change in the solution temperature during the repeated cycling (Figure 2H). The UV-Vis-NIR absorbance values of the CDs:Gd,Dy-TAT@Exo-RGD solution at 808 nm showed no obvious changes under laser irradiation for 1 h. (Figure S9). All the results indicated that the CDs:Gd,Dy-TAT@Exo-RGD possessed satisfactory photothermal effects and high photothermal stability under laser irradiation, making them promising therapeutic agents for the treatment of tumors.

### 3.3. Tumor Cell Targeting, Nucleus Penetration, Cytotoxicity and the Photothermal Induced Cell Death In Vitro

**Figure 3:**
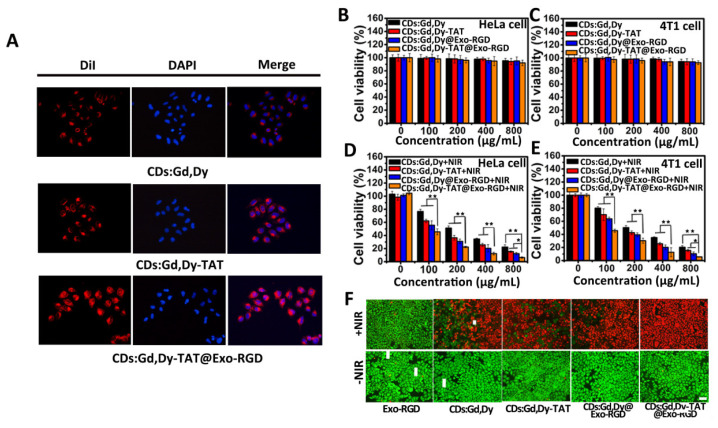
In vitro tumor cell targeting, nucleus penetration, cytotoxicity and photothermal performance. (**A**) DiI dye (red fluorescence)-labeled CDs:Gd,Dy, CDs:Gd,Dy-TAT or CDs:Gd,Dy-TAT@Exo-RGD was incubated with HeLa cells for 24 h, and DAPI was applied to dye the nucleus. The cell uptake was photographed through a fluorescence microscope, scale bar: 30 µm. (**B**–**E**) Viabilities of HeLa cells and 4T1 cells co-incubated with CDs:Gd,Dy, CDs:Gd,Dy-TAT, CDs:Gd,Dy@Exo-RGD or CDs:Gd,Dy-TAT@Exo-RGD of different concentrations (CDs:Gd,Dy: 0, 100, 200, 400 and 800 μg·mL^−1^) with/without exposure to laser (1.6 W·cm^−2^, 8 min). (**F**) Fluorescence images of Exo-RGD, CDs:Gd,Dy, CDs:Gd,Dy-TAT, CDs:Gd,Dy@Exo-RGD or CDs:Gd,Dy-TAT@Exo-RGD treated HeLa cells stained with Calcein AM and PI before and after laser irradiation (1.6 W·cm^−2^, 8 min), scale bar: 50 µm, * *p* < 0.05, ** *p* < 0.01.

The TAT peptide was beneficial to cell nucleus targeting, and the RGD peptide modification made it efficient for tumor cell targeting. Therefore, it was believed that the CDs:Gd,Dy-TAT@Exo-RGD could act as a dual-ligand targeting drug delivery nanoplatform towards tumors; the targeting was tested through intracellular uptake experiments with recipient cells. The DiI (a red fluorescence dye) labeled CDs:Gd,Dy, CDs:Gd,Dy-TAT and CDs:Gd,Dy-TAT@Exo-RGD were incubated with HeLa cells for 24 h. The uptake of these NPs was detected by fluorescence microscope (Figure 3A). For HeLa cells co-incubated with CDs:Gd,Dy, no evident red fluorescence was observed, demonstrating that few CDs:Gd,Dy were taken up by the cells. For the cells treated with CDs:Gd,Dy-TAT, the fluorescence intensity was dramatically increased compared to CDs:Gd,Dy. Stronger and brighter red fluorescence was found from the cells incubated with CDs:Gd,Dy-TAT@Exo-RGD, and most of the red fluorescence was located in the nucleus region, which indicated that the CDs:Gd,Dy-TAT@Exo-RGD could efficiently accumulate at cell nuclei. CDs:Gd,Dy@Exo-RGD without TAT peptide was mainly located in the cytoplasm, demonstrating the nuclei targeting action of the TAT peptide (Figure S10). These results displayed that the CDs:Gd,Dy-TAT@Exo-RGD could selectively accumulate in tumor cells with deep nuclear-permeating performance. The in vitro cytotoxicity of CDs:Gd,Dy-TAT@Exo-RGD was tested by the MTT method on HeLa cells and 4T1 cells. After 24 h co-incubation with CDs:Gd,Dy, CDs:Gd,Dy-TAT, CDs:Gd,Dy@Exo-RGD or CDs:Gd,Dy-TAT@Exo-RGD, the HeLa and 4T1 cells viabilities were all greater than 90% when the concentrations were 800 μg·mL^−1^ (Figure 3B,C), showing that the NPs presented superior biocompatibility without laser irradiation. The photothermally induced cell death under NIR laser irradiation of CDs:Gd,Dy-TAT@Exo-RGD on HeLa and 4T1 cells were also measured via the MTT method. As depicted in Figure 3D,E, the cell viabilities significantly decreased with concentration increase when exposed to NIR laser. It should be noted that the cell viabilities of the CDs:Gd,Dy-TAT@Exo-RGD (800 μg·mL^−1^) treated cells were less than 10% for both HeLa cells and 4T1 cells, which were lower than that of CDs:Gd,Dy (~24%), CDs:Gd,Dy-TAT (~18%) and CDs:Gd,Dy@Exo-RGD (~ 13%).

The photothermal efficiency of CDs:Gd,Dy-TAT@Exo-RGD was further investigated through Calcein-AM (green fluorescence for live cells) and PI (red fluorescence for dead cells) dying. As shown in Figure 3F, when incubated with Exo-RGD, CDs:Gd,Dy, CDs:Gd,Dy-TAT, CDs:Gd,Dy@Exo-RGD or CDs:Gd,Dy-TAT@Exo-RGD without irradiation, the cells showed strong bright green fluorescence and no apparent red fluorescence. As displayed in Figure S11, no clear red fluorescence was found when the concentration of CDs:Gd,Dy-TAT@Exo-RGD was as high as 800 μg·mL^−1^. Once the NIR laser irradiation was conducted (Figure 3F), the Exo-RGD treated cells still showed strong green fluorescence, while most of the CDs:Gd,Dy, CDs:Gd,Dy-TAT and CDs:Gd,Dy@Exo-RGD treated cells showed red fluorescence. It should be noted that nearly all the cells showed red fluorescence when treated with CDs:Gd,Dy-TAT@Exo-RGD. In order to exclude disturbance from the laser, the HeLa cells were irradiated with NIR laser alone; the bright green fluorescence (Figure S12) revealed that the laser alone could not cause cell death, thus, the photothermal effect resulted from CDs:Gd,Dy. In all, CDs:Gd,Dy-TAT@Exo-RGD could accumulate at the tumor cells with an increased concentration compared to CDs:Gd,Dy and CDs:Gd,Dy-TAT, and showed high photothermal conversion efficiency and excellent biosafety properties, which make it an ideal photothermal agent for cancer.

### 3.4. Tumor Targeting of CDs:Gd,Dy-TAT@Exo-RGD In Vivo

**Figure 4:**
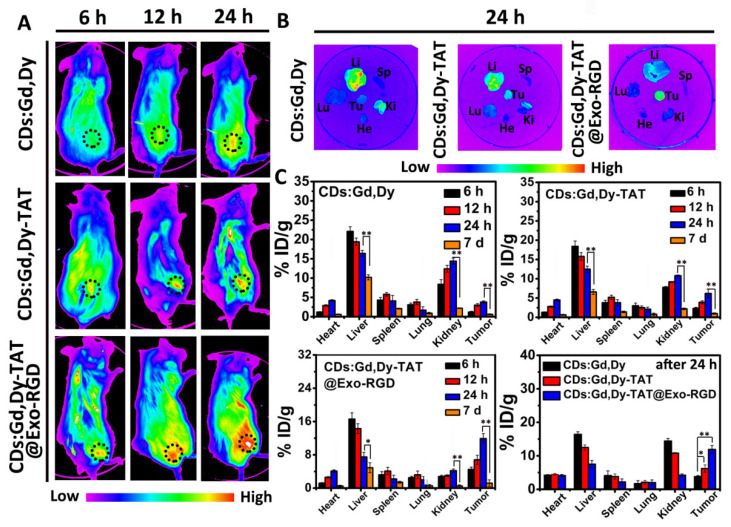
In vivo tumor targeting of CDs:Gd,Dy-TAT@Exo-RGD. (**A**) Fluorescence images of tumor-bearing mice intravenously injected with CDs:Gd,Dy, CDs:Gd,Dy-TAT or CDs:Gd,Dy-TAT@Exo-RGD at different time points; the tumor site is circled with dark dots. (**B**) Ex vivo fluorescence images of major organs (heart: He, liver: Li, spleen: Sp, lung: Lu, kidney: Ki) and tumor (Tu) collected from mice for different treatment post 24 h. (**C**) Biodistribution analysis of mice for different treatment at different time points through compared the Gd concentration measured by ICP-710ES, * *p* < 0.05, ** *p* < 0.01.

Tumor-bearing mice were used to study the tumor targeting capacity and biodistribution of CDs:Gd,Dy-TAT@Exo-RGD in vivo. Fluorescence probe Cy5.5 labeled CDs:Gd,Dy, CDs:Gd,Dy-TAT or CDs:Gd,Dy-TAT@Exo-RGD was injected into the mice through the tail vein, then the mice were monitored using a noninvasive small animals living imager. Without TAT and Exo-RGD encapsulation, only slight fluorescence was found at the tumor site post 24 h injection (Figure 4A). In sharp contrast, we detected strong fluorescence at the tumor site for the mice injected with CDs:Gd,Dy-TAT@Exo-RGD, and the fluorescence signal was enhanced over time, reaching its maximum at 24 h. Next, the mice were sacrificed and the tumor and main organs were collected for ex vivo imaging. As shown in Figure 4B, consistent with the in vitro imaging results, there was a strong fluorescence signal at the tumor site from the CDs:Gd,Dy-TAT@Exo-RGD treated mice, while fluorescence signals from the mice treated with CDs:Gd,Dy were mainly observed in the liver and kidney rather than the tumor site. This indicates that CDs:Gd,Dy modified with TAT and encapsulated into Exo-RGD showed improved targeting ability to tumor and prolonged blood circulation time in vivo.

To characterize the tumor targeting ability of CDs:Gd,Dy-TAT@Exo-RGD more accurately, a biodistribution analysis was conducted by measuring the Gd concentration. After intravenous injection with CDs:Gd,Dy, CDs:Gd,Dy-TAT and CDs:Gd,Dy-TAT@Exo-RGD at different time intervals, the tumors and main organs were collected for ICP-710ES to measure Gd concentration. As shown in Figure 4C, most of the CDs:Gd,Dy accumulated in the liver and kidney via the mononuclear phagocytes of RES [37,38], while little was located in tumor tissue. For the mice injected with CDs:Gd,Dy-TAT, the majority of Gd was located in the liver and kidney, with little at the tumor site. By contrast, the highest Gd accumulation in tumor sites was observed from the mice injected with CDs:Gd,Dy-TAT@Exo-RGD. The content of Gd increased over time and reached its peak at 24 h, which was inconsistent with the imaging results both in vitro and in vivo. It was found that the Gd concentrations in the heart and lung were relatively low at all times post-injection for all treated groups. Even though there was still some Gd accumulated in the liver after 24 h, this accumulation was considered to cause negligible side-effect in vivo, as the previous experiments had showed that the CDs:Gd,Dy-TAT@Exo-RGD had excellent biosafety. Moreover, the Gd content was significantly decreased after 7 d administration, avoiding long-term toxicity. The preferential accumulation of CDs:Gd,Dy-TAT@Exo-RGD at deep tumor sites guaranteed the further PTT effect under laser irradiation.

### 3.5. MRI and CT Imaging of Tumor In Vitro and In Vivo

**Figure 5:**
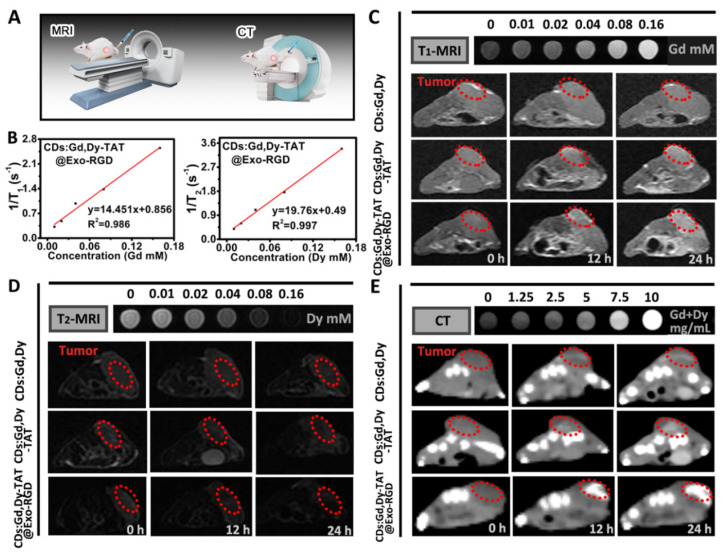
*T*_1_-MRI, *T*_2_-MRI and CT imaging performance of CDs:Gd,Dy-TAT@Exo-RGD. (**A**) The schematic illustration of MRI and CT imaging. (**B**) *r*_1_/*r*_2_ relaxivity values of CDs:Gd,Dy-TAT@Exo-RGD. In vivo *T*_1_-MRI (**C**), *T*_2_-MRI (**D**) and CT imaging (**E**) of tumor-bearing mice before and after intravenous injection of CDs:Gd,Dy, CDs:Gd,Dy-TAT or CDs:Gd,Dy-TAT@Exo-RGD for 12 h and 24 h; the red dots circle the tumor site and the images at the top are the corresponding phantom images.

Imaging techniques can detect tumor locations, tumorigenesis and metastasis, and realize the maximum of therapeutic efficiency in the process of therapy. Theoretically, CDs:Gd,Dy could act as both a *T*_1_-MRI and *T*_2_-MRI contrast agent due to the doped rare earth elements Gd and Dy. To verify the imaging performance of CDs:Gd,Dy-TAT@Exo-RGD, a 3.0 T clinical MRI system was introduced to conduct MR imaging (Figure 5A). For quantitative evaluation of the imaging performance, the longitudinal relaxivity value (*r*_1_) and the transverse relaxivity value (*r*_2_) were measured to be 14.45 mM^−1^·s^−1^ and 19.76 mM^−1^·s^−1^, higher than that of clinical Gd-DTPA (3.69 mM^−1^·s^−1^) [39] (Figure 5B). The enhanced relaxivity value of the CDs:Gd,Dy-TAT@Exo-RGD was mainly by reason of its excellent water solubility. Additionally, the TAT peptide modification and Exo-RGD encapsulation showed nearly no influence on relaxivity performance in vitro, as there was no distinct change of *r*_1_ and *r*_2_ value between CDs:Gd,Dy, CDs:Gd,Dy-TAT and CDs:Gd,Dy-TAT@Exo-RGD (Figure S13). In Figure 5C,D, both the *T*_1_-MRI and *T*_2_-MRI signals of the phantom pictures were gradually enhanced with the increase of Gd/Dy concentration in vitro. As for the in vivo *T*_1_-MRI and *T*_2_-MRI, the tumor-bearing mice were injected with CDs:Gd,Dy, CDs:Gd,Dy-TAT and CDs:Gd,Dy-TAT@Exo-RGD (15 mg Gd/Dy·kg^−1^·wt) via the tail vein, and the mice were imaged using the same clinical MRI scanner. *T*_1_-MRI images became distinctly brighter and *T*_2_-MRI images became obviously darker at the tumor sites as time progressed. Not surprisingly, the strongest contrast was observed in the CDs:Gd,Dy-TAT@Exo-RGD treated mice because of the dual-ligand targeting effect and exosomes encapsulation.

Due to the strong X-ray absorption capacity of Gd and Dy, the CDs:Gd,Dy-TAT@Exo-RGD NPs were expected to act as CT imaging contrast agents. Firstly, the phantom images of CDs:Gd,Dy-TAT@Exo-RGD solutions were conducted with a clinical CT instrument. The images displayed an obvious concentration-dependent contrast enhancement (Figure 5E). An excellent linearity was found in CT value (HU) and the Gd/Dy concentration, with a slope of 48.6 HU·L·g^−1^ (Figure S14), higher than that of Iobitridol (a commercial CT contrast agent) at equivalent concentrations [40]. Furthermore, the tumor-bearing mice were intravenously injected with CDs:Gd,Dy, CDs:Gd,Dy-TAT or CDs:Gd,Dy-TAT@Exo-RGD solution for 12 h and 24 h to conduct in vivo CT imaging (Figure 5E and Figure S15). The CT images exhibited distinct bright signals at tumor sites, especially for the CDs:Gd,Dy-TAT@Exo-RGD group. The signal intensity increased with time extension and reached its maximum after 24 h. All the results indicated that the dual-ligand targeting and exosomes encapsulation were effective at deeply delivering the NPs to the tumor site, and the superior MRI and CT imaging performance of CDs:Gd,Dy-TAT@Exo-RGD would be beneficial to guide photothermal therapy in vivo.

### 3.6. In Vivo PTT Effect of CDs:Gd,Dy-TAT@Exo-RGD

**Figure 6:**
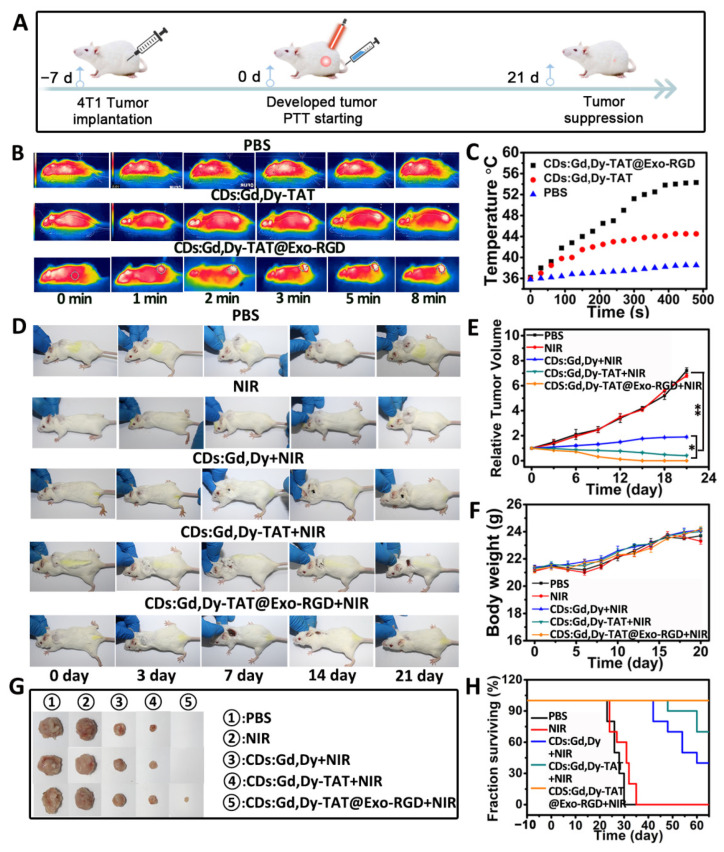
In vivo tumor PTT effect of CDs:Gd,Dy-TAT@Exo-RGD. (**A**) The schematic illustration of photothermal therapy in vivo. Thermal infrared images (**B**) and the temperature change (**C**) of tumor in tumor-bearing mice injected with PBS, CDs:Gd,Dy-TAT or CDs:Gd,Dy-TAT@Exo-RGD followed by NIR laser irradiation (1.6 W·cm^−2^, 8 min). Representative mice photographs (**D**), relative tumor volumes (**E**) and body weights (**F**) of the mice from the PBS, NIR, CDs:Gd,Dy+NIR, CDs:Gd,Dy-TAT+NIR and CDs:Gd,Dy-TAT@Exo-RGD+NIR groups. (**G**) Photographs of tumors collected from different groups at the end of the treatment. (**H**) Survival rate of the mice after different treatments (* *p* < 0.05, ** *p* < 0.01).

Encouraged by the in vitro photothermal efficacy and in vivo multimodal imaging results, we evaluated the photothermal therapy effect of CDs:Gd,Dy-TAT@Exo-RGD on mice (Figure 6A). The biodistribution analysis identified enhanced accumulation of CDs:Gd,Dy-TAT@Exo-RGD into tumors. therefore, we firstly conducted photothermal imaging through a thermal infrared camera in order to visualize the temperature variation of the tumors in real-time. The tumor-bearing mice were injected with PBS, CDs:Gd,Dy-TAT or CDs:Gd,Dy-TAT@Exo-RGD, followed by NIR laser irradiation after 24 h (Figure 6B,C). The temperature at the tumor site rose only ~2 °C after 8 min irradiation in the PBS only group, indicating that the NIR light was safe for normal organs and skin. The tumor temperature rose to 44 °C for the mice treated with CDs:Gd,Dy-TAT. Predictably, there was a rapid temperature increase in the tumors in the mice treated with CDs:Gd,Dy-TAT@Exo-RGD; the temperature kept constant at about 56 °C, which was sufficient to induce irreversible damage to tumor cells [41]. This temperature increase thus further confirmed the superior photothermal conversion performance of CDs:Gd,Dy-TAT@Exo-RGD.

For the in vivo PTT, we randomly divided the tumor-bearing mice into five groups (PBS, PBS+NIR, CDs:Gd,Dy+NIR, CDs:Gd,Dy-TAT+NIR and CDs:Gd,Dy-TAT@Exo-RGD+NIR). The mice were injected with the NPs through the tail vein, and the tumor sites were exposed to 808 nm NIR laser. The tumors in the PBS group and NIR group grew quickly as time passed, indicating that NIR laser irradiation alone failed to inhibit tumor growth. The growth rate of tumors in the CDs:Gd,Dy+NIR group was lower than the other two groups (Figure 6D,E). There was little inhibition effect on tumor growth with CDs:Gd,Dy-TAT+NIR treatment. In contrast, an obvious tumor suppression effect was detected in the CDs:Gd,Dy-TAT@Exo-RGD+NIR group, where the tumors began to shrink at day 3 and were completely ablated after 21 days. These results validated the excellent targeting ability and enhanced PTT efficacy of CDs:Gd,Dy-TAT@Exo-RGD. During the therapeutic period, as exhibited in Figure 6F, none of the mice showed evident weight variation. The mice were sacrificed after 21 days and the tumors were imaged to provide insight into the anti-tumor efficiency of the different treatments (Figure 6G). The mice from the PBS or PBS+NIR group were all dead in succession within 30 days. The survival rate of mice treated with CDs:Gd,Dy-TAT@Exo-RGD+NIR after 60 days was 100%, which was higher than those of the CDs:Gd,Dy-TAT+NIR (40%) and CDs:Gd,Dy-TAT@Exo-RGD+NIR (70%) groups (Figure 6H). These encouraging results proved that our prepared CDs:Gd,Dy-TAT@Exo-RGD could be a satisfactory candidate as a PTT agent for tumor therapy.

### 3.7. In Vivo Systematic Biocompatibility Assessment

**Figure 7:**
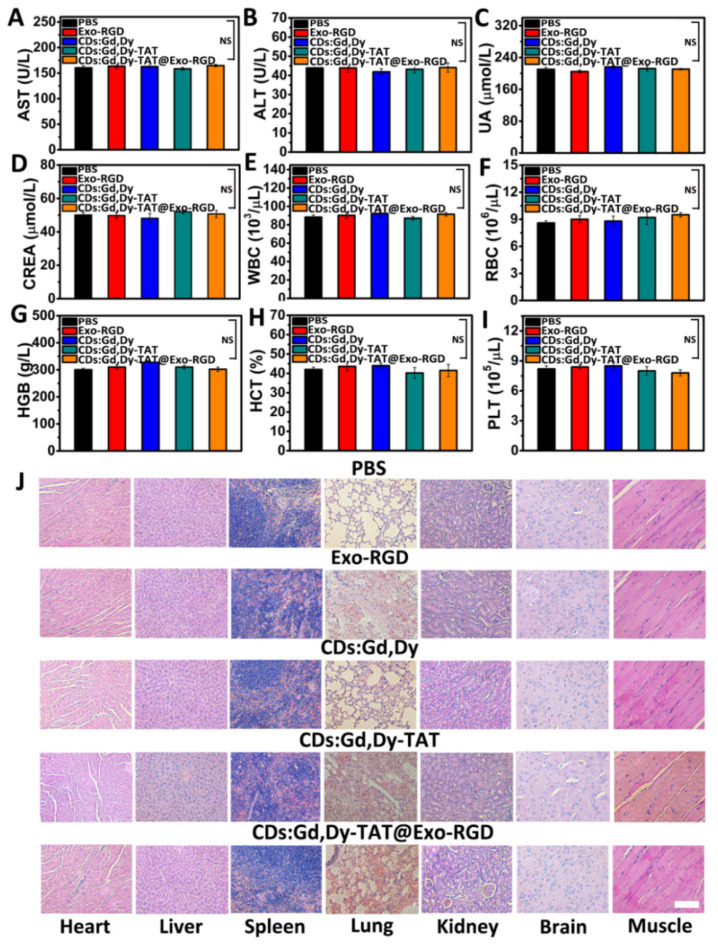
In vivo biocompatibility assessment. (**A**–**D**) Serum biochemical markers of ALT, AST, UA, and CREA and (**E**–**I**) whole blood cell analysis of the mice treated with PBS, Exo-RGD, CDs:Gd,Dy, CDs:Gd,Dy-TAT or CDs:Gd,Dy-TAT@Exo-RGD two weeks following administration, NS, *p* > 0.05. (**J**) H&E images of major organs (heart, liver, spleen, lung, and kidney), brain and muscle from different treatment mice, scale bar: 100 µm.

The toxicity and biocompatibility of CDs:Gd,Dy-TAT@Exo-RGD were systematically assessed to ensure its further clinical translation. Healthy mice were randomly divided into five groups followed by intravenous injection of PBS, Exo-RGD CDs:Gd,Dy, CDs:Gd,Dy-TAT or CDs:Gd,Dy-TAT@Exo-RGD. After feeding for two weeks, the main organs (heart, liver, spleen, lung and kidney) and blood were collected after sacrificing the treated mice. Blood biochemical analysis was conducted to test the serum parameters including alanine aminotransferase (ALT), aspartic acid aminotransferase (AST), creatinine (CREA) and uric acid (UA). The levels of AST and ALT in blood may reflect liver function; liver injury occurs when AST and ALT levels are elevated [42]. As depicted in Figure 7A,B, AST and ALT were all in normal range, indicating that there was no significant liver toxicity. The main parameters that reflect kidney function (CREA and UA) were similar to those of healthy mice (Figure 7C,D) [43]. The results demonstrated that there was not any abnormality in the liver and kidney after treatment with the NPs. In addition, the complete blood parameters including red blood cells (RBC), white blood cells (WBC), hemoglobin (HGB), platelets (PLT) and hematocrit (HCT) were also measured (Figure 7E−I). There was no distinct difference in any of the groups compared to the healthy mice. Moreover, similar results were also found for the tumor-bearing mice treated with CDs:Gd,Dy-TAT@Exo-RGD+NIR at the end of treatment (Figures S16 and S17). The main organs from the above groups were dyed with H&E. As displayed in Figure 7J, there was no distinct inflammation and toxicity in any of the treated groups. The morphology and structure of the cells were observed to be normal and there was no necrosis in any of the samples. For the mice treated with CDs:Gd,Dy-TAT@Exo-RGD+NIR, at the end of treatment, there was also no abnormality, as observed by H&E staining (Figure S18). All of the results demonstrated that the CDs:Gd,Dy-TAT@Exo-RGD showed high in vivo biocompatibility without obvious side effects. Combined with its excellent imaging ability and photothermal efficacy for tumors, our prepared CDs:Gd,Dy-TAT@Exo-RGD is a promising nanoplatform for imaging guided tumor therapy.

## 4. Conclusions

Exosomes for drug delivery and tumor therapy possess the ability to overcome biological barriers, long blood circulation time, and low immunogenicity; however, there are still several limitations that need to be settled, such as insufficient accumulation in tumors and the unsatisfactory imaging performance. Herein, we have successfully developed a multiple targeted delivery nanoplatform of engineered exosomes-mediated carbon dots (CDs:Gd,Dy-TAT@Exo-RGD) for imaging guided cancer therapy. The in vitro and in vivo experiments demonstrate that CDs:Gd,Dy-TAT@Exo-RGD can target tumor cells effectively and penetrate deeply into cell nuclei. Under NIR laser irradiation, CDs:Gd,Dy-TAT@Exo-RGD can ablate tumors with minimal side effects. Most importantly, both the MRI/CT imaging effect and tumor therapy effect of CDs:Gd,Dy-TAT@Exo-RGD were enhanced compared with the CDs:Gd,Dy and CDs:Gd,Dy-TAT groups. This work presented an outstanding exosomes nanoplatform with high tumor theranostic efficiency.

## Data Availability

Not applicable.

## Supplementary Materials

The following are available online at https://www.mdpi.com/article/10.3390/pharmaceutics13101593/s1, Figure S1: Representative TEM images of CDs:Gd,Dy-TAT. Figure S2: RGD-FITC absorbance–concentration standard curve at 490 nm. Figure S3: TAT-FITC absorbance–concentration standard curve at 490 nm. Figure S4: Hydrodynamic diameter of CDs:Gd,Dy-TAT@Exo-RGD in water, PBS, DMEM and FBS after incubation for 48 h. Figure S5: (a) Possible Gd^3+^ and Dy^3+^ release from CDs:Gd,Dy-TAT@Exo-RGD in FBS solution after dialysis for 48 h after NIR laser irradiation, using XO as Gd^3+^ and Dy^3+^ indicator. (b) Possible Gd^3+^ and Dy^3+^ release from GdCl_3_ and DyCl_3_ solution after dialysis for 48 h, which was set as control. (A: blank XO, B: 0.2 mg·mL^−1^, C: 0.4 mg·mL^−1^, D: 0.6 mg·mL^−1^, E: 0.8 mg·mL^−1^, F: 1.0 mg·mL^−1^). Figure S6: Possible Gd^3+^ and Dy^3+^ release from CDs:Gd,Dy-TAT@Exo-RGD in FBS solution after dialysis for 48 h before and after NIR laser irradiation, using XO as Gd^3+^ and Dy^3+^ indicator (A: blank XO, B: NPs before NIR laser, C:NPs after NIR laser). Figure S7: Possible Gd^3+^ (a) and Dy^3+^ (b) release from CDs:Gd,Dy-TAT@Exo-RGD in FBS solution after dialysis, for different time before and after NIR laser irradiation. (A: dialysis for 48 h before NIR, B: dialysis for 72 h before NIR, C: dialysis for 48 h after NIR, D: dialysis for 72 h after NIR); rare earth elements were measured by ICP-710ES. The overall Gd^3+^ and Dy^3+^ content in the FBS solution were also measured. Figure S8: TEM image of CDs:Gd,Dy-TAT@Exo-RGD after NIR laser irradiation. Figure S9: UV-Vis-NIR absorption value of CDs:Gd,Dy@Exo-RGD solution at 808 nm under laser irradiation for 1 h. Figure S10: DiI dye-labeled CDs:Gd,Dy@Exo-RGD were co-cultured with HeLa cells for 24 h, and the nuclei of the cells were stained with DAPI. The cells were visualized using fluorescence microscope, scale bar: 30 µm. Figure S11: Fluorescence images of different concentrations (CDs:Gd,Dy: 0, 100, 200, 400 and 800 μg·mL^−1^) of CDs:Gd,Dy-TAT@Exo-RGD treated HeLa cells after laser irradiation (1.6 W·cm^−2^, 8 min). The cells were stained with Calcein AM and PI before imaging, scale bar: 100 µm. Figure S12: Fluorescence images of HeLa cells treated with laser irradiation only (1.6 W·cm^−2^, 8 min). The cells were stained with Calcein AM and PI before imaging, scale bar: 100 µm. Figure S13: *T*_1_ (a−c) and *T*_2_ (d−f) relaxation times as a function of different Gd and Dy concentrations in CDs:Gd,Dy (a,d), CDs:Gd,Dy-TAT (b,e) and CDs:Gd,Dy-TAT@Exo-RGD (c,f). Figure S14: HU values of CDs:Gd,Dy-TAT@Exo-RGD solution at various concentrations of Gd and Dy (1.25, 2.5, 5, 7.5 and 10 mg·Gd and Dy·mL^−1^). Figure S15: HU values of tumor sites before and after intravenous injection of CDs:Gd,Dy, CDs:Gd,Dy-TAT or CDs:Gd,Dy-TAT@Exo-RGD solution for 12 h and 24 h. Figure S16: Serum biochemical marker analysis of ALT (a), AST (b), UA (c), and CREA (d) of mice from CDs:Gd,Dy-TAT@Exo-RGD+NIR treated group at the end of the PTT treatment. Healthy mice were set as control, NS, *p* > 0.05. Figure S17: Whole blood cell counts of mice from CDs:Gd,Dy-TAT@Exo-RGD+NIR treated group at the end of the PTT treatment. Healthy mice were set as control, NS, *p* > 0.05. Figure S18: H&E stained images of brain, muscle and major organs (heart, liver, spleen, lung, and kidney) of mice from CDs:Gd,Dy-TAT@Exo-RGD+NIR treated group at the end of treatment, scale bar: 100 µm.
