# Supplementary material for: Engineered Exosomes-Based Photothermal Therapy with MRI/CT Imaging Guidance Enhances Anticancer Efficacy through Deep Tumor Nucleus Penetration
Source: Pharmaceutics. 2021 Sep 30;13(10):1593. doi: 10.3390/pharmaceutics13101593 (PMC8538523; doi:10.3390/pharmaceutics13101593)
Supplement: Supplementary file 1 [file pharmaceutics-13-01593-s001.zip › pharmaceutics-1357256-supplementary-update.pdf]

pharmaceutics

MDPI

Supplementary Materials: Engineered Exosomes-Based Photothermal Therapy with MRI/CT Imaging Guidence Enhances Anticancer Efficacy through Deep Tumor Nucleus Penetration

Min Yang, Xiaohui Wang, Fang Pu, Ying Liu, Jia Guo, Shuzhuo Chang, Guoying Sun, and Yinghua Peng

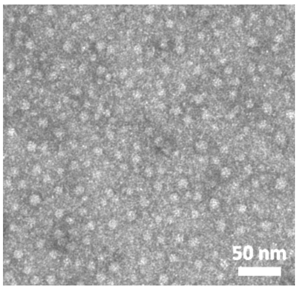

Figure S1. Representative TEM images of CDs:Gd,Dy-TAT.

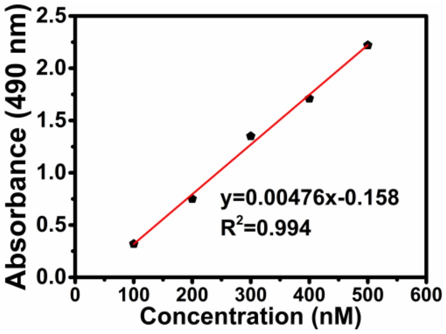

Figure S2. RGD-FITC absorbance-concentration standard curve at 490 nm.

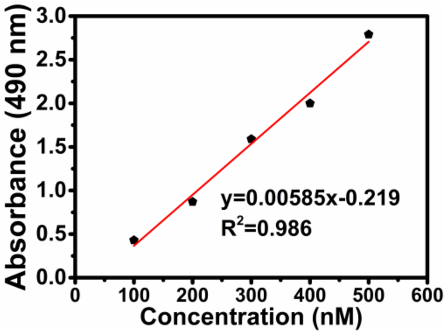

Figure S3. TAT-FITC absorbance-concentration standard curve at 490 nm.

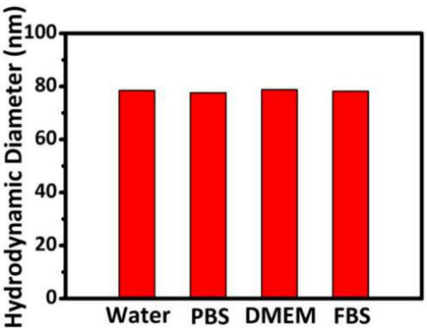

Figure S4. Hydrodynamic diameter of CDs:Gd,Dy-TAT@Exo-RGD in water, PBS, DMEM and FBS after incubation for 48 h.

Gd^{3+} and Dy^{3+} leakage assay

Since free Gd^{3+} is high toxicity, the possible rare earth element Gd/Dy release from the CDs:Gd,Dy-TAT@Exo-RGD solution into surroundings was determined using xylenol orange as indicator or measured by ICP-710ES. In a typical experiment, 0.5 mL CDs:Gd,Dy-TAT@Exo-RGD (before and after laser irradiation) was mixed with 10 mL FBS. The solution was added into a dialysis bag with a MWCO of 2000, then the bag was placed in a tube, which contained 25 mL FBS. Next, the tube was shaken on a magnetic stirrer at the speed of 100 rpm at 37 °C. 200 μL of the dialyzate was collected from the tube after 48 h. The xylenol orange acetic buffer solution was prepared: 3.0 mg xylenol orange was dissolved with 200 mL of acetate buffer (pH = 5.8). Next, 200 μL xylenol orange solution and 100 μL dialyzate were mixed evenly. $GdCl_3$ and $DyCl_3$ solutions at the equivalent concentration were chosen as control. In order to validate the results from xylenol orange measurement, Gd^{3+} and Dy^{3+} concentrations were acquired by ICP-710ES as well, the detection limit of which is 10^{-3} μg·mL^{-1}.

Results: CDs:Gd,Dy-TAT@Exo-RGD was dispersed in water, PBS, DMEM and FBS to analyse its stability. There was no visible precipitation after 48 h incubation. The diameter obtained from DLS remained minimum change for the CDs:Gd,Dy-TAT@Exo-RGD (Figure S4). According to previous report, free Gd^{3+} can inhibit Ca ions channel resulting in cardiovascular toxicity and neurotoxicity [1]. Hence, detecting the possible Gd^{3+} and Dy^{3+} leakage was necessary to assess the physiological stability and toxicity of CDs:Gd,Dy-TAT@Exo-RGD. The strong chelation of rare earth and carboxyl is looking forward to prevent the leakage of Gd^{3+} and Dy^{3+} into external surrounding. To test this assumption, different concentrations of CDs:Gd,Dy-TAT@Exo-RGD solutions were dispersed in FBS followed by dialysis. Then the xylenol orange (XO) was used as indicator to detect the possible Gd^{3+} and Dy^{3+} leakage, setting $GdCl_3$ and $DyCl_3$ solution as control. When the Gd^{3+} concentration was as low as 0.125 mM, the XO can still react with free Gd^{3+} to form red complex [2]. The color of the dialyzate remained unchanged when the concentration was reach up to 1.0 mg·mL^{-1}, illustrating that our prepared CDs:Gd,Dy-TAT@Exo-RGD couldn't react with XO (Figure S5 and S6). Furthermore, we used ICP-710ES to measure the possible Gd^{3+} leakage, which had much higher sensitivity. As shown in Figure S7, we observed no rare earth element leakage both before and after laser irradiation. These results showed that the CDs:Gd,Dy-TAT@Exo-RGD had great physiological stability.

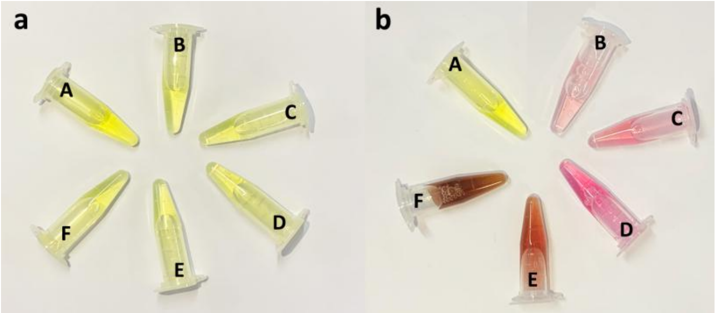

Figure S5. (**a**) Possible Gd^{3+} and Dy^{3+} release from CDs:Gd,Dy-TAT@Exo-RGD in FBS solution after dialysis for 48 h after NIR laser irradiation using XO as Gd^{3+} and Dy^{3+} indicator. (**b**) Possible Gd^{3+} and Dy^{3+} release from $GdCl_3$ and $DyCl_3$ solution after dialysis for 48 h, which was setting as control. (A: blank XO, B: 0.2 mg·mL^{-1}, C: 0.4 mg·mL^{-1}, D: 0.6 mg·mL^{-1}, E: 0.8 mg·mL^{-1}, F: 1.0 mg·mL^{-1}).

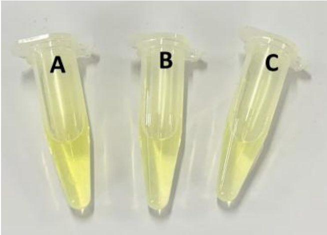

Figure S6. Possible Gd^{3+} and Dy^{3+} release from CDs:Gd,Dy-TAT@Exo-RGD in FBS solution after dialysis for 48 h before and after NIR laser irradiation using XO as Gd^{3+} and Dy^{3+} indicator (A: blank XO, B: NPs before NIR laser, C: NPs after NIR laser).

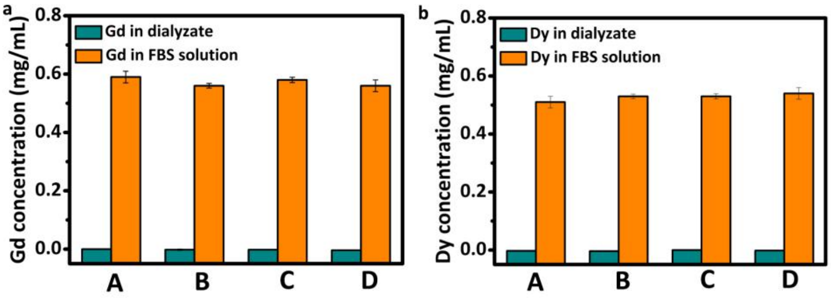

Figure S7. Possible Gd^{3+} (**a**) and Dy^{3+} (**b**) release from CDs:Gd,Dy-TAT@Exo-RGD in FBS solution after dialysis for different time before and after NIR laser irradiation. (A: dialysis for 48 h before NIR, B: dialysis for 72 h before NIR, C: dialysis for 48 h after NIR, D: dialysis for 72 h after NIR) The rare earth element was measured by ICP-710ES. The overall Gd^{3+} and Dy^{3+} content in the FBS solution were also measured.

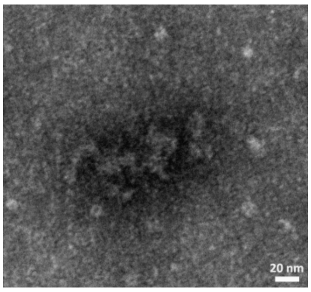

Figure S8. TEM image of CDs:Gd,Dy-TAT@Exo-RGD after NIR laser irradiation.

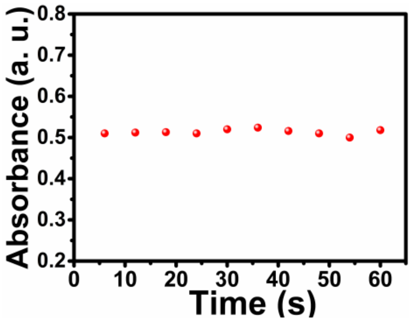

Figure S9. UV-Vis-NIR absorption value of CDs:Gd,Dy@Exo-RGD solution at 808 nm under laser irradiation for 1 h.

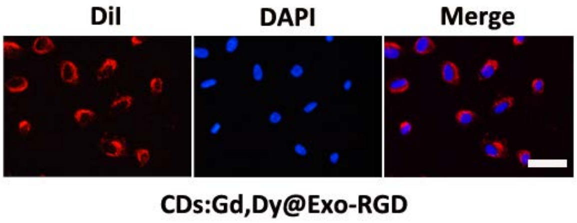

Figure S10. DiI dye-labeled CDs:Gd,Dy@Exo-RGD were co-cultured with HeLa cells for 24 h, and the nucleic of the cells were stained with DAPI. The cells were visualized using fluorescence microscope, scale bar: 30 μm.

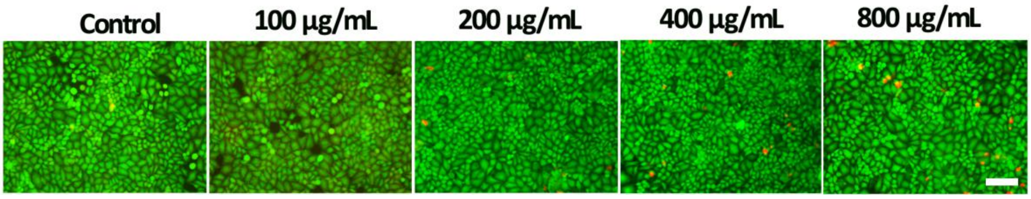

Figure S11. Fluorescence images of different concentrations (CDs:Gd,Dy: 0, 100, 200, 400 and 800 $\mu g \cdot mL^{-1}$) of CDs:Gd,Dy-TAT@Exo-RGD treated HeLa cells after laser irradiation (1.6 $W \cdot cm^{-2}$, 8 min). The cells were stained with Calcein AM and PI before imaging, scale bar: 100 μm.

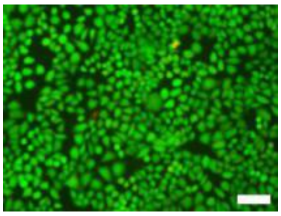

Figure S12. Fluorescence images of HeLa cells treated with laser irradiation only (1.6 $W \cdot cm^{-2}$, 8 min). The cells were stained with Calcein AM and PI before imaging, scale bar: 100 μm.

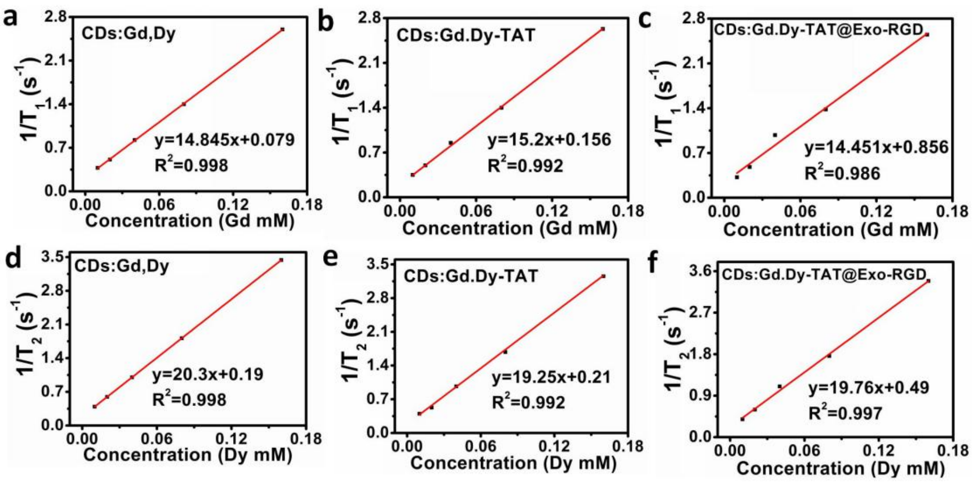

Figure S13. T_1 (**a**-**c**) and T_2 (**d**-**f**) relaxation times as a function of different Gd and Dy concentrations in CDs:Gd,Dy (**a**,**d**), CDs:Gd,Dy-TAT (**b**,**e**) and CDs:Gd,Dy-TAT@Exo-RGD (**c**,**f**).

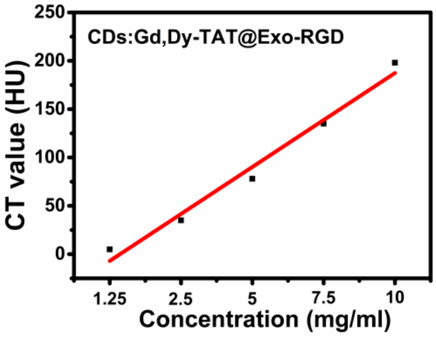

Figure S14. HU values of CDs:Gd,Dy-TAT@Exo-RGD solution at various concentrations of Gd and Dy (1.25, 2.5, 5, 7.5 and 10 mg·Gd and Dy·mL^{-1}).

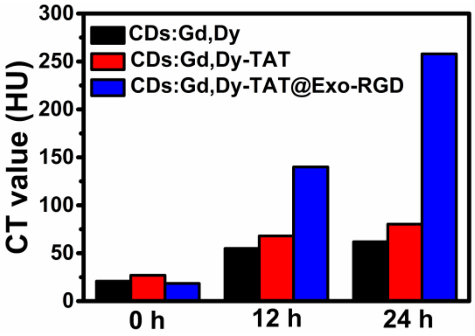

Figure S15. HU values of tumor sites before and after intravenous injection of CDs:Gd,Dy, CDs:Gd,Dy-TAT or CDs:Gd,Dy-TAT@Exo-RGD solution for 12 h and 24 h.

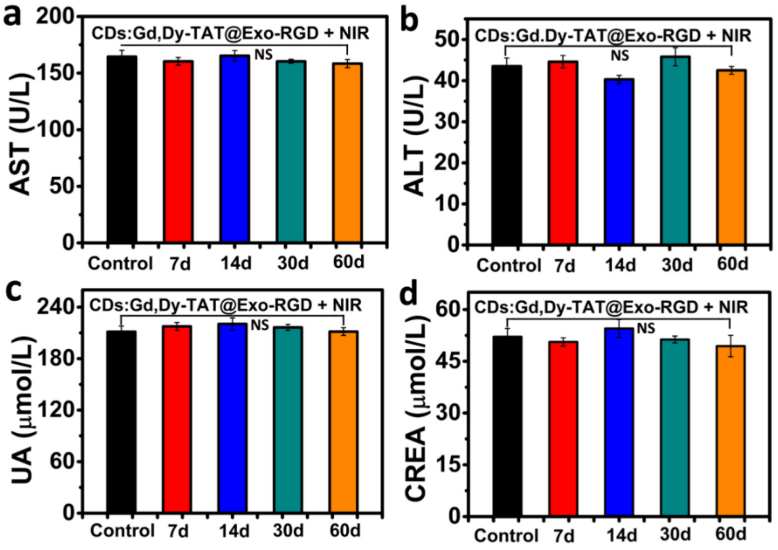

Figure S16. Serum biochemical marker analysis of ALT (**a**), AST (**b**), UA (**c**), and CREA (**d**) of mice from CDs:Gd,Dy-TAT@Exo-RGD+NIR treated group at the end of the PTT treatment. Healthy mice were set as control, NS, $p > 0.05$.

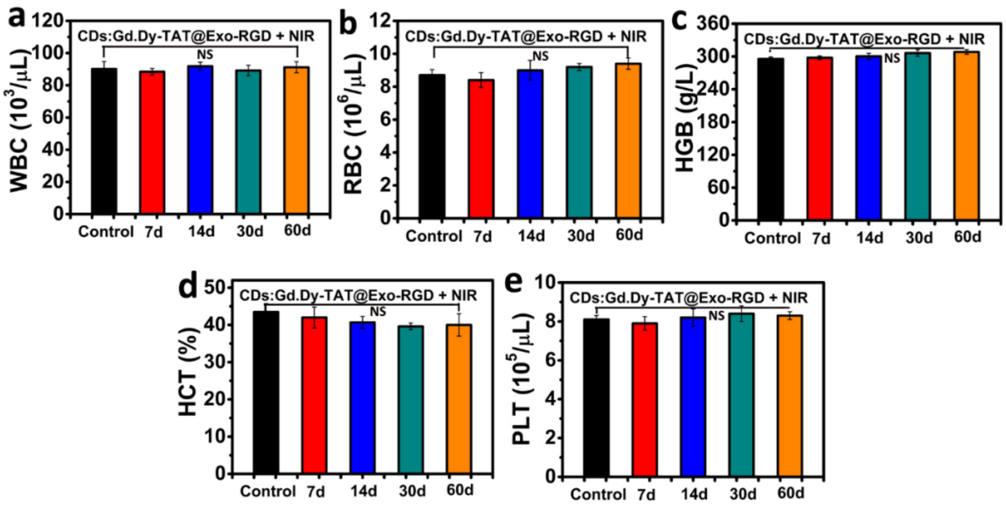

Figure S17. Whole blood cell counts of WBC (**a**), RWC (**b**), HGB (**c**), HCT (**d**) and PLT (**e**) of.mice from CDs:Gd,Dy-TAT@Exo-RGD+NIR treated group at the end of the PTT treatment. Healthy mice were set as control, NS, $p > 0.05$.

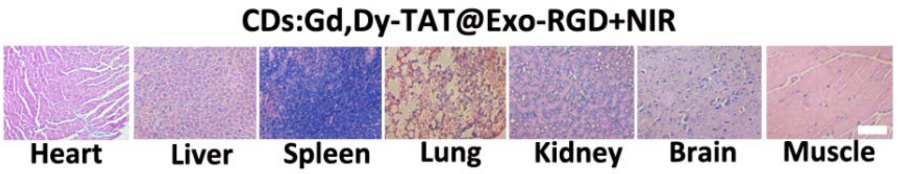

Figure S18. H&E stained images of brain, muscle and major organs (heart, liver, spleen, lung, and kidney) of mice from CDs:Gd,Dy-TAT@Exo-RGD+NIR treated group at the end of treatment, scale bar: 100 μm.

References

1. H. Chen, G.D. Wang, W. Tang, T. Todd, Z. Zhen, C. Tsang, K. Hekmatyar, T. Cowger, R.B. Hubbard, W. Zhang, J. Stickney, B. Shen, J. Xie, Gd-Encapsulated Carbonaceous Dots with Efficient Renal Clearance for Magnetic Resonance Imaging, *Adv. Mater.*, **2014**, 26, 6761–6766.
2. Y. Shi, Y. Pan, J. Zhong, J. Yang, J. Zheng, J. Cheng, R. Song, C. Yi, Facile synthesis of gadolinium (III) chelates functionalized carbon quantum dots for fluorescence and magnetic resonance dual-modal bioimaging, *Carbon*, **2015**, 93, 742–750.
